# The Impact of Semen Exposure on the Immune and Microbial Environments of the Female Genital Tract

**DOI:** 10.3389/frph.2020.566559

**Published:** 2020-11-09

**Authors:** Janine Jewanraj, Sinaye Ngcapu, Farzana Osman, Andile Mtshali, Ravesh Singh, Leila E. Mansoor, Salim S. Abdool Karim, Quarraisha Abdool Karim, Jo-Ann S. Passmore, Lenine J. P. Liebenberg

**Affiliations:** ^1^Center for the AIDS Programme of Research in South Africa (CAPRISA), Durban, South Africa; ^2^Department of Medical Microbiology, School of Laboratory Medicine and Medical Science, University of KwaZulu-Natal, Durban, South Africa; ^3^Department of Microbiology, National Health Laboratory Services, KwaZulu-Natal Academic Complex, Inkosi Albert Luthuli Central Hospital, Durban, South Africa; ^4^School of Nursing and Public Health, University of KwaZulu-Natal, Durban, South Africa; ^5^Department of Epidemiology, Columbia University, New York, NY, United States; ^6^Institute of Infectious Diseases and Molecular Medicine (IDM), University of Cape Town, Cape Town, South Africa; ^7^National Health Laboratory Services, Johannesburg, South Africa

**Keywords:** Y-chromosome DNA, semen, genital inflammation, HIV, cytokines, microbes, matrix metalloproteinases, immune cells

## Abstract

**Background:** Semen induces an immune response at the female genital tract (FGT) to promote conception. It is also the primary vector for HIV transmission to women during condomless sex. Since genital inflammation and immune activation increase HIV susceptibility in women, semen-induced alterations at the FGT may have implications for HIV risk. Here we investigated the impact of semen exposure, as measured by self-reported condom use and Y-chromosome DNA (YcDNA) detection, on biomarkers of female genital inflammation associated with HIV acquisition.

**Methods:** Stored genital specimens were collected biannually (mean 5 visits) from 153 HIV-negative women participating in the CAPRISA 008 tenofovir gel open-label extension trial. YcDNA was detected in cervicovaginal lavage (CVL) pellets by RT-PCR and served as a biomarker of semen exposure within 15 days of genital sampling. Protein concentrations were measured in CVL supernatants by multiplexed ELISA, and the frequency of activated CD4+CCR5+ HIV targets was assessed on cytobrush-derived specimens by flow cytometry. Common sexually transmitted infections (STIs) and bacterial vaginosis (BV)-associated bacteria were measured by PCR. Multivariable linear mixed models were used to assess the relationship between YcDNA detection and biomarkers of inflammation over time.

**Results:** YcDNA was detected at least once in 69% (106/153) of women during the trial (median 2, range 1–5 visits), and was associated with marital status, cohabitation, the frequency of vaginal sex, and Nugent Score. YcDNA detection but not self-reported condom use was associated with elevated concentrations of several cytokines: IL-12p70, IL-10, IFN-γ, IL-13, IP-10, MIG, IL-7, PDGF-BB, SCF, VEGF, β-NGF, and biomarkers of epithelial barrier integrity: MMP-2 and TIMP-4; and with reduced concentrations of IL-18 and MIF. YcDNA detection was not associated with alterations in immune cell frequencies but was related to increased detection of *P. bivia* (OR = 1.970; CI 1.309–2.965; *P* = 0.001) at the FGT.

**Conclusion:** YcDNA detection but not self-reported condom use was associated with alterations in cervicovaginal cytokines, BV-associated bacteria, and matrix metalloproteinases, and may have implications for HIV susceptibility in women. This study highlights the discrepancies related to self-reported condom use and the need for routine screening for biomarkers of semen exposure in studies of mucosal immunity to HIV and other STIs.

## Introduction

In sub-Saharan Africa, women account for the majority of Human Immunodeficiency Virus (HIV) infections compared to their male counterparts (1) and remain a key target population for the development of biomedical HIV prevention strategies. The risk of HIV infection in young women is increased in the context of genital inflammation (2, 3), and efforts to better understand the causes of inflammation at the female genital tract (FGT) may inform on the design of targeted approaches to prevent HIV acquisition. HIV requires access to local cellular targets at the FGT to establish productive infection, and cytokine biomarkers of genital inflammation may be linked to HIV risk through their role in cellular recruitment (4). Furthermore, genital cytokine concentrations are also associated with alterations in the integrity of the vaginal epithelium (4), and with the abundance of bacterial vaginosis (BV)-associated microbes at the FGT (5–7), both implicated in susceptibility to HIV infection.

Sex without a condom remains the primary mode of HIV-1 transmission, with semen acting as the major vector for male to female transmission of the virus (8). Semen consists of several pro- and anti-inflammatory factors and functions as a biological modifier at the FGT to facilitate pregnancy and conception (9–11). Semen exposure has been associated with temporary upregulation of cytokines and the recruitment of leukocytes to the cervical epithelium and stroma (9–14). A pro-inflammatory immune response is generally mounted against semen in the FGT, resulting in the removal of excess and abnormal sperm (15, 16). Semen also contains a diverse array of microbial communities and has an alkaline pH, all of which have the potential to alter the vaginal microbiome (17–20). Apart from the immune altering capacity of semen itself, sexual intercourse has been associated with a significant reduction in *Lactobacillus crispatus* (17), increased prevalence of *Gardnerella vaginalis* (21), and may also lead to vaginal epithelial microabrasions (22, 23) that facilitate HIV entry and access to local target cells at the female genital mucosa. These alterations at the FGT may have implications for the risk of HIV acquisition in women.

Semen-associated inflammation may be, however, short-lived, as immune tolerance to paternal alloantigens is induced during reproduction (9, 11, 24, 25). Semen contains anti-inflammatory compounds such as transforming growth factor-β which promotes a shift from a type 1 helper (Th1) to a type 2 helper (Th2) immune response at the FGT, thereby inducing a regulatory T cell (Treg) response (14, 16, 25). Semen also contains high concentrations of prostaglandin E2, which has been shown to inhibit macrophage cytokine production and T cell proliferation (25–27). These anti-inflammatory responses responsible for tolerance to sperm may also inhibit the control of pathogens such as HIV and other sexually transmitted infections (STIs) at the FGT. Taken together, efforts to prevent HIV infection may benefit from a better understanding of the contribution that both pro- and anti-inflammatory properties of semen have on the risk of HIV acquisition in women.

Self-reported condom use is often used as an indication of semen exposure at the FGT. However, this practice is subject to bias, and data are often misreported (28–30). Routine objective screening for the presence of semen biomarkers as opposed to self-reports of condom use may be useful to reliably assess the frequency of condomless sex e.g., during HIV prevention trials, to assess mucosal immunity to STIs, and to further characterize the impact of semen on the FGT in the context of HIV. Y-chromosome DNA (YcDNA) detection in female genital specimens has previously been used as a reliable biomarker of semen exposure within 15 days of sampling (31–37). Y-chromosome polymerase chain reaction (PCR) is a highly stable, sensitive, and specific method to detect spermatozoa-associated deoxyribonucleic acid (DNA) fragments of the sex-determining region and testis-specific protein Y-encoded (TSPY) genes of the Y-chromosome that are not present on the X-chromosome gene (36, 38–41). Considering the established unreliability of self-reported condom use, we hypothesized that YcDNA detection, but not self-reported condom use will be associated with alterations in biomarkers of inflammation linked to HIV risk in women.

## Methodology

### Study Design and Population

This longitudinal retrospective study included questionnaire data and stored genital samples from 153 randomly-selected HIV negative women from the CAPRISA 008 trial (42). The CAPRISA 008 trial was an open-label extension trial to assess the effectiveness of delivering tenofovir 1% gel in the context of routine family planning services (42). The women enrolled in this study were aged 20–44 years old, were from urban and rural KwaZulu-Natal, and had previously participated in the parent CAPRISA 004 efficacy trial (43). At the time of initial sampling, all participants had not used 1% tenofovir gel for a minimum of 3 years since exiting the CAPRISA 004 trial and were subsequently provided the tenofovir gel for use throughout the CAPRISA 008 trial, supplied either through CAPRISA clinic sites (control arm) or through family planning services (intervention arm). Genital specimens were collected every 6 months during the 2-year trial period (average 5 ± 1 visits). All participants of the CAPRISA 008 trial provided informed consent for the storage of their specimens for use in future studies (BFC237/010). This study was approved by the Biomedical Research Ethics Committee at the University of KwaZulu-Natal under the ethics number BE258/19. YcDNA detection was conducted at the Medical Microbiology Department at the University of KwaZulu-Natal, and all other laboratory assays were conducted at the CAPRISA Mucosal Immunology Laboratory in Durban, South Africa.

### Specimen Collection and Processing

Genital specimens including cervical cytobrushes, cervicovaginal lavage (CVL), and vaginal swabs were collected from the participants at each biannual visit. The collection and processing of CVL specimens was previously reported by Bebell et al. (44). Briefly, a plastic bulb pipette was inserted toward the cervical os through a lubricated speculum. A volume of 5 ml sterile saline was inserted and allowed to bathe the cervix. The resulting fluid accumulated at the posterior fornix and was collected using the same pipette and dispensed into a sterile conical tube. Thereafter the CVL specimens were transported to the CAPRISA laboratory. At the laboratory the specimens were centrifuged, and the supernatant was removed and stored in 1 ml aliquots at −80°C.

Cervical cytobrush specimens were collected as previously reported (45). Briefly, a Digene cervical sampler was used to collect cervical mononuclear cells from all participants under speculum examination. The cytobrush was inserted into the endocervical canal and gently rotated 360° to collect cells from the cervical os. The cytobrush specimens were placed into a sterile 15 ml tube (Griener) containing transport medium [Roswell Park Memorial Institute Medium 1640 (Sigma-Aldrich) supplemented with 10% heat-inactivated Fetal Bovine Serum and 5 mM glutamine, penicillin, and streptomycin]. Any specimen containing visible blood was discarded.

Vaginal swabs were collected from the posterior fornices and lateral vaginal walls of each participant and tested for the presence STIs and BV-associated bacteria.

### Human Y-Chromosome Detection Assay (PrimerDesign Ltd, UK)

Total DNA was extracted from stored CVL pellet specimens using the MagNAPure LC DNA Isolation Kit I (Roche Applied Science, Indianapolis, IN), according to the manufacturer's instructions. A region of the TSPY1 gene on the Y-chromosome was amplified using the Applied Biosystems® QuantStudio™ 5 RT-PCR System (Thermo Fisher Scientific). YcDNA concentrations were determined from a 1:4 standard curve dilution series. The amplification of the Y-chromosome within 36 cycles was considered a positive result. The negative control (containing no DNA) and an extraction control (PrimerDesign Ltd, UK) were included in each run. Detection of the Y-chromosome and analysis of the results was performed as outlined in the manufacturer's protocol (PrimerDesign Ltd, UK). YcDNA is reported to be stable in the FGT for up to 15 days after sex (31–33) and served as a biomarker of semen exposure in this study.

### Quantification of Soluble Protein Biomarkers of Inflammation in Genital Fluid

Concentrations of 48 cytokines, 9 matrix metalloproteinases (MMPs), and 4 tissue inhibitors of metalloproteinases (TIMPs) were measured in undiluted CVL supernatant specimens, according to the manufacturer's instructions. The concentrations of each analyte was measured using the Bio-Plex Pro Human Cytokine, MMP, and TIMP kits and a Bio-Plex Array Reader (Bio-Rad Laboratories) as previously reported (3). The cytokine panel included interleukin (IL)-1α, IL-1β, IL-2, IL-3, IL-4, IL-5, IL-6, IL-7, IL-8, IL-9, IL-10, IL-12p40, IL-12p70, IL-13, IL-15, IL-16, IL-17, IL-18, IL-1 receptor antagonist (IL-1RA), IL-2 receptor α (IL-2Rα), cutaneous T cell attracting chemokine (CTACK), growth related oncogene (GRO)-α, hepatocyte growth factor (HGF), interferon (IFN)-γ, IFN-α2, leukemia inhibitory factor (LIF), monocyte chemotactic protein (MCP)-3, macrophage migration inhibitory factor (MIF), monokine induced by gamma interferon (MIG), β-nerve growth factor (NGF), stem cell factor (SCF), stem cell growth factor (SCGF)-β, stromal cell-derived factor (SDF)-1α, tumor necrosis factor (TNF)-α, TNF-β, TNF-related apoptosis-inducing ligand (TRAIL), fibroblast growth factor (FGF)-basic, eotaxin, granulocyte colony-stimulating factor (G-CSF), granulocyte-macrophage (GM)-CSF, macrophage (M)-CSF, interferon gamma-induced protein (IP)-10, MCP-1, macrophage inflammatory protein (MIP)-1α, MIP-1β, platelet-derived growth factor BB (PDGF-BB), regulated on activation, normal T cell expressed and secreted (RANTES) and vascular endothelial growth factor (VEGF). The MMP and TIMP panels included MMP-1, MMP-2, MMP-3, MMP-7, MMP-8, MMP-9, MMP-10, MMP-12, MMP-13, TIMP-1, TIMP-2, TIMP-3, and TIMP-4. Cytokine data were available for all visits (*n* = 679), while MMP/TIMP data was only generated at baseline (*n* = 145, Supplementary Figure 1). The sensitivity of these kits ranged between 0.2 and 45.4 pg/ml for the cytokines and between 1 and 450 pg/ml for each of the MMPs measured in this study. Data collection was conducted using the Bio-Plex Manager software version 6. Sample protein concentrations were calculated from standard curves using a five-parameter logistic regression formula. Cytokine and MMP concentrations below the lower limit of detection were reported as half of the minimum concentration measured for each analyte. Likewise, concentrations above the detectable limit were recorded as double the maximum concentration measured for each analyte. To reduce the impact of inter-plate variability, all CVL specimens collected from each participant over time were run on the same assay plate. Intra-plate and inter-plate variability were assessed to detect significant differences between duplicate or inter-plate wells, respectively, and Spearman rho ≥ 0.8, and non-significant *p-*values were considered acceptable.

### STI and Microbe Detection

Vaginal swab specimens were used for STI and microbe detection at the National Health Laboratory Services, Inkosi Albert Luthuli Central Hospital Academic Complex (46). Multiplex PCR amplification was performed on the ABI® 7500 platform from Applied Biosystems (Thermo Fisher Scientific) and using the FTD (Fast-track diagnostics) STD9 kit according to the manufacturer's instructions. The kit contained primers and TaqMan probes that were designed from highly conserved regions of genetic sequences for pathogens associated with STIs, namely *Neisseria gonorrhoeae, Chlamydia trachomatis, Trichomonas vaginalis, Gardnerella vaginalis, Mycoplasma genitalium*, and Herpes simplex virus (HSV)-1/2. Concentrations of two Lactobacilli strains, *Lactobacillus crispatus* and *Lactobacillus jensenii* (Assay ID Ba04646245_s1, Ba04646258_s1) and BV-associated bacteria i.e., *Gardnerella vaginalis, Prevotella bivia*, BVAB2, and *Atopobium vaginae* (Assay ID Ba04646236_s1, Ba04646278_s1, Ba04646229_s1, Pa04646150_s1, respectively) were measured using Applied Biosystems™ TaqMan® assays. All reactions were run on an ABI® 7500 platform from Applied Biosystems (Thermo Fisher Scientific) RT-PCR machine. STI data was available for all visits (*n* = 676), while data on BV-associated bacteria was available for all visits but baseline (*n* = 534, Supplementary Figure 1). Gram stain microscopy was used to assess for BV by Nugent Score (47). Women were diagnosed as negative, intermediate, or having BV (Nugent Score 0–3, 4–6, and 7–10, respectively).

### Investigation of Immune Cell Frequency

Cervical cytobrush specimens were used to measure the dynamics and frequency of activated (CD38+ or HLA-DR+) or replicating (Ki67+) T cells (CD3+CD4+ or CD3+CD8+) and CD4+CCR5+ targets for HIV replication using multiparametric flow cytometry. Data acquisition was conducted using a LSRII flow cytometer (BD Immunocytometry Systems) and analyzed using FlowJo Software version 9.9 (Tree Star, C, US). Gates differentiating negative and positive populations were set by fluorescence minus one staining. Specimens with a cervical CD3+ T cell event count below 100 were excluded from the analysis. The gating strategy is represented in Supplementary Figure 2.

### Statistical Considerations

The Shapiro-Wilk normality test was conducted to determine the distribution of the data. The Mann-Whitney *U-*test was used to compare continuous variables, and the Fisher's exact test was used to compare proportions between the groups at baseline. Questionnaire data were available for 146 participants at baseline, and linear regression models were used to investigate the relationship between self-reported condom use (always vs. never) and biomarkers of inflammation [cytokine concentrations (pg/ml), MMP/TIMP concentrations (pg/ml) and immune cell frequencies (%)] at baseline. Soluble protein concentrations were log10-transformed and immune cell frequencies were converted to proportions to ensure normality. Additionally, linear mixed models accounting for repeated measures were used to assess the relationships between YcDNA detection and cytokine concentrations and immune cell frequencies over time. A generalized estimating equation (GEE) model using a logit link and accounting for repeated measures was used to determine the impact of semen exposure on vaginal microbe presence over time. The unadjusted models controlled for study arm, i.e., CAPRISA or family planning services, and time in the study. Multivariable models were adjusted for variables associated with inflammation or HIV risk such as study arm, time in study, Nugent Score, participant age, presence of STIs, the number of vaginal sex acts in the last 30 days, and genital inflammation status. Genital inflammation status was defined by the median cytokine concentration across all visits for each participant in the upper quartile of the distribution of cytokine concentrations (as calculated using the entire dataset) (2). Given that genital inflammation is a linear combination of cytokines, this variable was not controlled for in cytokine analyses. *P*-values were adjusted for multiple comparisons using the Benjamini-Hochberg method. All tests were conducted at the 5% level of significance. Statistical analyses were performed using GraphPad Prism version 8.3.1 (GraphPad Software, San Diego, CA), STATA version 15.0 (StataCorp., College Station, Texas, USA), and SAS version 9.4 (SAS Institute Inc., Cary, NC, USA).

## Results

### Baseline Characteristics of the Study Population

Demographic data was available for 95% (146/153) of all women at baseline. Overall, the median age of the population was 28 years [interquartile range [IQR] 25–33 years; Table 1], with 39% of women having detectable YcDNA in their genital fluid at baseline (57/146 women). More women with detectable YcDNA were married (24.6 vs. 10.1%, *P* = 0.038), living with their partner (33.3 vs. 16.9%, *P* = 0.027), and reported seeing their partner more often (36.8 vs. 21.6%, *P* = 0.017) than those without detectable YcDNA. Additionally, YcDNA detection was associated with a higher median number of lifetime pregnancies [median 2 (IQR 1–3) vs. median 1 (IQR 1–2), respectively, *P* = 0.042], and the number of vaginal sex acts in the 30 days prior to sampling [median 5 (IQR 3–10) vs. median 4 (IQR 2–6), respectively, *P* = 0.008]. Of the women reporting to have always used a condom during sex, 31% (17/54) had detectable YcDNA in their vaginal specimens, highlighting the discrepancies related to self-reported condom use. Gonorrhoeae detection was significantly associated with YcDNA detection (8.8 vs. 0%, respectively, *P* = 0.009). Women with detectable YcDNA also had a higher median Nugent Score [median 3 (IQR 1–7) vs. median 1 (IQR 0–3), respectively, *P* = 0.006].

**Table 1:** Baseline participant characteristics by YcDNA detection in female genital specimens.

**Characteristics**	**Level**	**Overall (*N =* 146)**	**YcDNA+ (*N =* 57)**	**YcDNA– (*N =* 89)**	**P-Value**
Age (years)	Median (IQR)	28 (25–33)	29 (25–35)	28 (25–30)	0.632
Educational level [% (n)]	Primary School	39.0% (57)	43.9% (25)	36.0% (32)	0.060
	HS complete	54.1% (79)	56.1% (32)	52.8% (47)	
	Tertiary complete	4.8% (7)	0	7.9% (7)	
	Less than primary	2.1% (3)	0	3.4% (3)	
Relationship status [% (n)]	Married	15.8% (23)	24.6% (14)	10.1% (9)	**0.038**
	Stable partner	82.9% (121)	73.7% (42)	88.8% (79)	
	Casual Partner	1.4% (2)	1.8% (1)	1.1% (1)	
Study arm [% (n)]	Intervention	47.3% (69)	50.9% (29)	44.9% (40)	0.502
	Control	52.7% (77)	49.1% (28)	55.1% (49)	
Age of regular/stable partner (years)	Median (IQR)	32 (28–37)	32 (28–38)	32 (29–36)	0.633
Number of lifetime pregnancies	Median (IQR)	2 (1–2)	2 (1–3)	1 (1–2)	**0.042**
Number of vaginal sex acts in the last 30 days	Median (IQR)	4 (2–8)	5 (3–10)	4 (2–6)	**0.008**
Partner HIV status [% (n)]	Positive	2.1% (3)	3.5% (2)	1.1% (1)	0.281
	Negative	65.8% (96)	70.2% (40)	62.9% (56)	
	Unknown	32.2% (47)	26.3% (15)	36.0% (32)	
Partner circumcision [% (n/N)]	Yes	32.8% (41/125)	27.5% (14/51)	36.5% (27/74)	0.542
	No	64.8% (81/125)	70.6% (36/51)	60.8% (45/74)	
	Unknown	2.4% (3/125)	2.0% (1/51)	2.7% (2/74)	
Partner living together [% (n)]	Yes	23.3% (34)	33.3% (19)	16.9% (15)	**0.027**
	No	76.7% (112)	66.7% (38)	83.1% (74)	
How often do you see regular partner [% (n/N)]	Daily	27.6% (40/145)	36.8% (21/57)	21.6% (19/88)	**0.017**
	Weekly	42.8% (62/145)	47.4% (27/57)	39.8% (35/88)	
	Monthly	26.9% (39/145)	14.0% (8/57)	35.2% (31/88)	
	< Monthly	2.8% (4/145)	1.8% (1/57)	3.4% (3/88)	
Contraceptive type [% (n)]	Depo-provera	57.5% (84)	63.2% (36)	53.9% (48)	0.105
	Oral contraceptive	21.9% (32)	17.5% (10)	24.7% (22)	
	Nur-isterate	14.4% (21)	8.8% (5)	18.0% (16)	
	Other	6.2% (9)	10.5% (6)	3.4% (3)	
Male condom use [% (n)]	Always	37.0% (54)	29.8% (17)	41.6% (37)	0.178
	Sometimes	49.3% (72)	50.9% (29)	48.3% (43)	
	Never	13.7% (20)	19.3% (11)	10.1% (9)	
HSV-2 antibodies [% (n)]	Positive	88.4% (129)	86.0% (49)	89.9% (80)	0.106
	Negative	9.6% (14)	8.8% (5)	10.1% (9)	
	Equivocal	2.1% (3)	5.3% (3)	0	
Human Papillomavirus [% (n)]	No	48.6% (71)	50.9% (29)	47.2% (42)	0.735
	Yes	51.4% (75)	49.1% (28)	52.8% (47)	
Any STIs [% (n/N)]	No	81.9% (118/144)	78.9% (45)	83.9% (73/87)	0.509
	Yes	18.1% (26/144)	21.1% (12)	16.1% (14/87)	
*Neisseria Gonorrhoeae*	No	96.5% (139/144)	91.2% (52)	100.0% (87/87)	**0.009**
	Yes	3.5% (5/144)	8.8% (5)	0	
*Chlamydia trachomatis*	No	93.1% (134/144)	93.0% (53)	93.1% (81/87)	1.000
	Yes	6.9% (10/144)	7.0% (4)	6.9% (6/87)	
*Trichomonas vaginalis*	No	95.1% (137/144)	96.5% (55)	94.3% (82/87)	0.704
	Yes	4.9% (7/144)	3.5% (2)	5.7% (5/87)	
*Mycoplasma genitalium*	No	95.8% (138/144)	94.7% (54)	96.6% (84/87)	0.681
	Yes	4.2% (6/144)	5.3% (3)	3.4% (3/87)	
Bacterial vaginosis [% (n/N)]	Median (IQR)	2 (0–4)	3 (1–7)	1 (0–3)	**0.006**
Negative	0–3	74.6% (106/142)	61.4% (35/57)	83.5% (71/85)	**0.001**
Intermediate	4–6	10.6% (15/142)	10.5% (6/57)	10.6% (9/85)	
BV	7–10	14.8% (21/142)	28.1% (16/57)	5.9% (5/85)	

*Significant P-values (P < 0.05) are indicated by bold font*.

### Biomarkers of Inflammation Were Not Distinguished by Self-Reported Condom Use

Linear regression models were used to investigate the reliability of self-reported condom use as a measure of semen exposure. Biomarkers of female genital inflammation were compared between women self-reporting always (*n* = 54) and never using a condom (*n* = 20) at their baseline visit. Multivariable linear regression models were adjusted for age, any STI, Nugent Score, the number of vaginal sex acts in the past 30 days, randomization arm, and inflammation status. Neither cytokine concentrations, MMP concentrations, nor immune cell frequencies differed between the groups after multivariable adjustments (Supplementary Tables 1–3, respectively).

### YcDNA Detection Was Associated With Alterations in Protein Biomarkers of Inflammation

**Figure 1:**
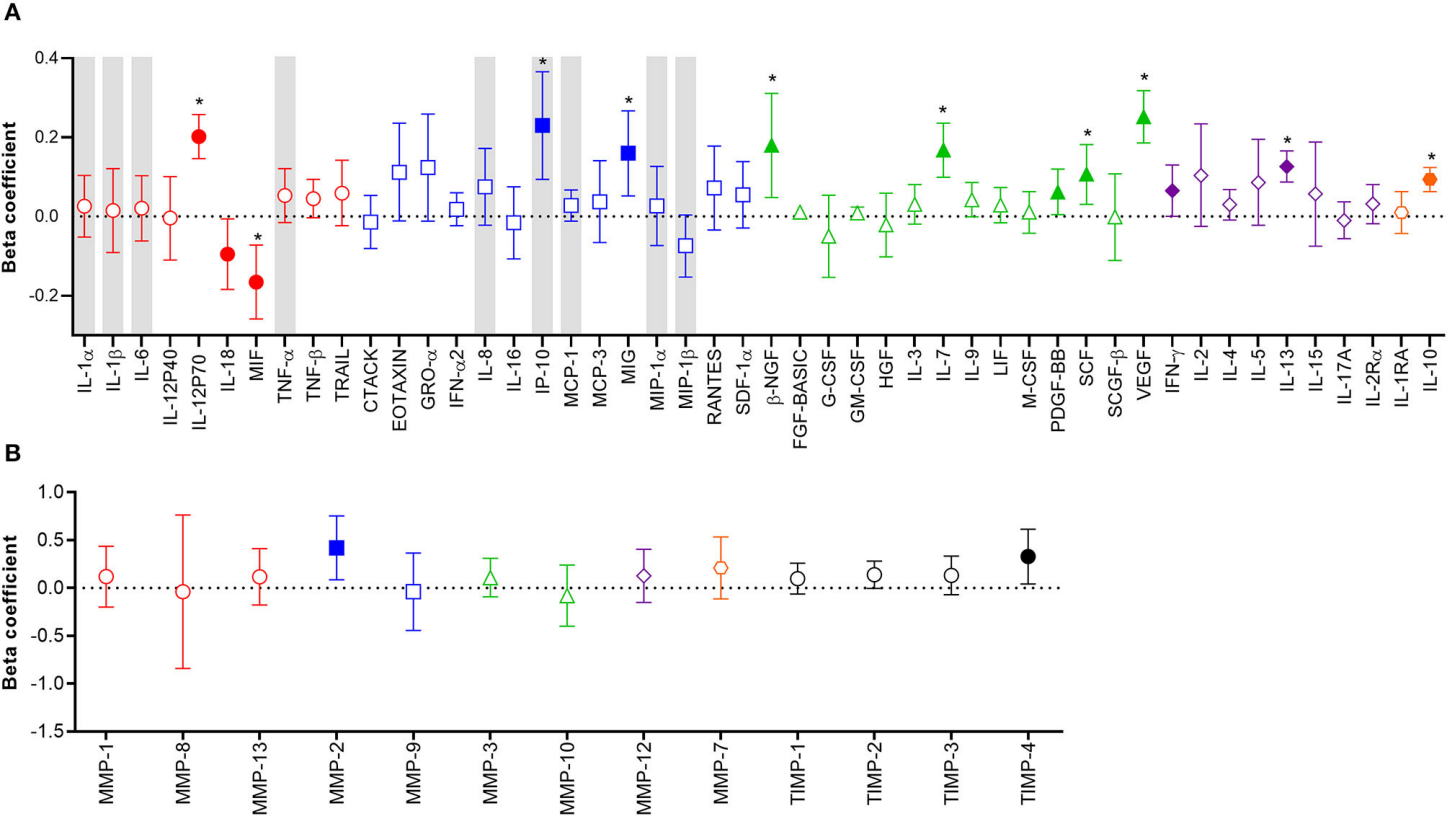
Association between protein biomarkers of inflammation and YcDNA detection in female genital specimens. β-coefficients and corresponding *P-*values for cytokine associations were determined using multivariable linear mixed models adjusting for age, any STI (*C. trachomatis, N. gonorrhoeae, T. vaginalis, and M. genitalium*), Nugent Score, number of vaginal sex acts in the past 30 days, randomization arm, and time in study. β-coefficients and corresponding *P*-values for MMP/TIMP associations were determined using multivariable linear regression models adjusting for age, any STI (*C. trachomatis, N. gonorrhoeae, T. vaginalis, and M. genitalium*), Nugent Score, number of vaginal sex acts in the past 30 days, randomization arm, and inflammation status. β-coefficients are depicted by shapes and error bars indicate the 95% CI. Significant *P*-values (*P* < 0.05) are indicated by filled symbols and significance after FDR adjustment is indicated by (*). **(A)** Cytokines are ordered according to functions: pro-inflammatory (red circles), chemotactic (blue squares), growth/haematopoiesis (green triangles), adaptive response (purple diamonds), and regulatory (orange hexagons) cytokines. Gray shadings represent the nine cytokines/chemokines previously associated with the definition of genital inflammation and/or in demonstrating its association with the risk of HIV infection (2, 3). **(B)** MMPs are grouped according to their functions: collagenases (red circles), gelatinases (blue squares), stromelysins (green triangles), macrophage elastase (purple diamond), matrilysin (orange hexagon), and TIMPs are represented by black circles.

Considering the potential unreliability in self-report of condom use, given that 31% of women who reported consistent condom use also had YcDNA evidence of recent condomless sex (Table 1), we determined whether a biomarker of semen exposure may be a better indicator of immune alterations at the FGT in response to semen. YcDNA detection within female genital specimens was used as a biomarker of semen exposure within 15 days prior to genital sampling (31–33). Linear mixed models were used to compare cytokine concentrations over time and linear regression models were used to compare MMP/TIMP concentrations at baseline between women with detectable YcDNA (semen exposure) and those without (no detectable semen exposure). Women with detectable YcDNA had significantly increased concentrations of IL-12p70 (β = 0.202; CI 0.146, 0.258; *P* < 0.001), IP-10 (β = 0.230; CI 0.094, 0.366; *P* = 0.001), MIG (β = 0.160; CI 0.052, 0.267; *P* = 0.004), β-NGF (β = 0.180; CI 0.048, 0.311; *P* = 0.008), IL-7 (β = 0.168; CI 0.099, 0.236; *P* < 0.001), PDGF-BB (β = 0.062; CI 0.005, 0.120; *P* = 0.035), SCF (β = 0.107; CI 0.031, 0.182; *P* = 0.006), VEGF (β = 0.252; CI 0.186, 0.318; *P* < 0.001), IFN-γ (β = 0.065; CI 0.000, 0.130; *P* = 0.049), IL-13 (β = 0.126; CI 0.087, 0.166; *P* < 0.001), IL-10 (β = 0.094; CI 0.063, 0.124; *P* < 0.001), and reduced concentrations of IL-18 (β = −0.095; CI −0.184, −0.006; *P* = 0.036) and MIF (β = –0.166; CI −0.259, −0.072; *P* = 0.001; Figure 1A) after adjusting for age, any STI, Nugent Score, the number of vaginal sex acts in the past 30 days, time in study, and randomization arm. These associations between YcDNA detection and concentrations of IL-12p70, MIF, IP-10, MIG, β-NGF, IL-7, SCF, VEGF, IL-13, and IL-10 remained significant even after false discovery rate (FDR) adjustments. The concentrations of MMPs and TIMPs were compared among women with detectable YcDNA and those without at baseline. YcDNA detection was associated with elevated concentrations of MMP-2 (β = 0.419; CI 0.084, 0.753; *P* = 0.015), and TIMP-4 (β = 0.328; CI 0.042, 0.614; *P* = 0.025; Figure 1B) after adjusting for age, any STI, Nugent Score, the number of vaginal sex acts in the past 30 days, inflammation status, and randomization arm.

### Increased Detection of BV-Associated Microbes at the FGT Linked to Semen Exposure

GEE models were used to determine whether semen exposure was linked to an increased presence of BV-associated microbes at the FGT. Women with detectable YcDNA had a significantly increased presence of *P. bivia* (OR=1.970; CI 1.309, 2.965; *P* = 0.001; Table 2) compared to those without, after adjusting for age, any STI, the number of vaginal sex acts in the past 30 days, inflammation status, time in study, and randomization arm. This association between YcDNA detection and increased presence of *P. bivia* maintained significance after FDR adjustments (*P* = 0.007).

**Table 2:** Comparison of vaginal microbes between women with and without detectable YcDNA.

**Microbe**	**OR (95% CI)**	***P*-Value**	**FDR**	**OR (95% CI)**	**Adj *P*-Value**	**FDR**
*L. crispatus*	1.083 (0.766–1.529)	0.653	0.653	1.082 (0.763–1.534)	0.659	0.659
*L. jensenii*	0.752 (0.514–1.099)	0.141	0.237	0.736 (0.506–1.070)	0.109	0.189
*A. vaginae*	0.666 (0.379–1.171)	0.158	0.237	0.647 (0.370–1.130)	0.126	0.189
BVAB2	1.141 (0.797–1.633)	0.472	0.566	1.136 (0.792–1.631)	0.489	0.586
*G. vaginalis*	1.427 (0.990–2.058)	0.057	0.171	1.362 (0.942–1.968)	0.100	0.189
*P. bivia*	1.954 (1.312–2.911)	**0.001**	**0.006**	1.970 (1.309–2.965)	**0.001**	**0.007**

*OR and 95% CI were determined using a GEE model with a logit link to account for repeated measures. The unadjusted model controlled for randomization arm and time. The adjusted model additionally controlled for age, the number of vaginal sex acts in the past 30 days, any STI (C. trachomatis, N. gonorrhoeae, T. vaginalis, M. genitalium), and inflammation status. Significant P-values (P < 0.05) are indicated by bold font*.

### The Presence of Semen Was Not Associated With Immune Cell Recruitment at the FGT

**Figure 2:**
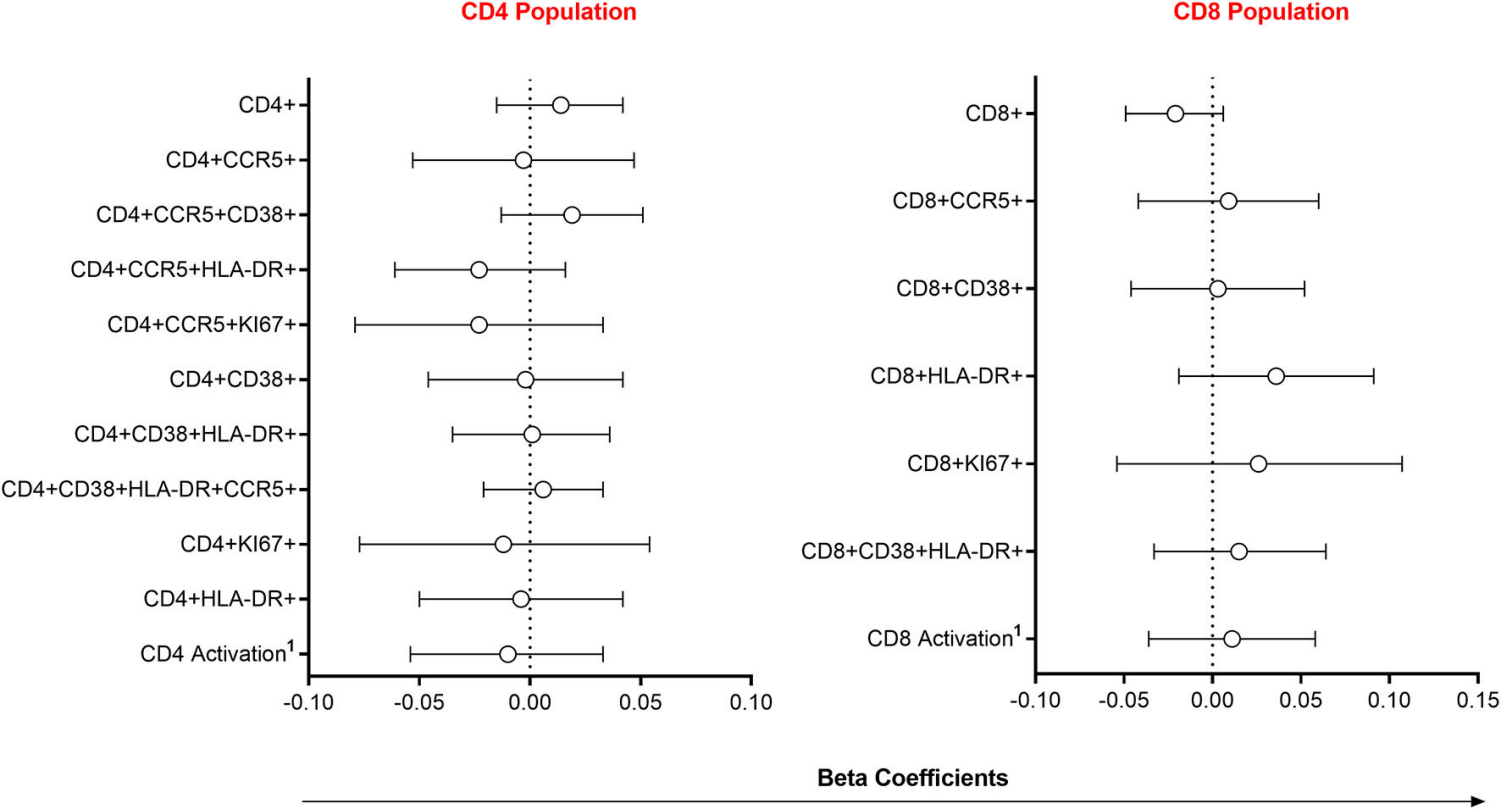
Association between immune cell frequencies and YcDNA detection in female genital specimens. β-coefficients and corresponding *P*-values were determined using multivariable linear mixed models adjusted for age, any STI (*C. trachomatis, N. gonorrhoeae, T. vaginalis, and M. genitalium*), Nugent Score, number of vaginal sex acts in the past 30 days, inflammation status, time in study, and randomization arm. β-coefficients are depicted by shapes and error bars indicate the 95% CI. ^1^Activation refers to cells expressing CCR5, HLA-DR and/or CD38.

Since alterations in mucosal cytokines and microbial microenvironments are associated with increased frequency of local HIV-susceptible cells (2, 6), we assessed the impact of semen exposure on the pool of available T cell targets at the FGT. Linear mixed models were used to compare immune cell frequencies between women with detectable YcDNA and those without. Immune cell frequencies were similar between women with detectable YcDNA in their vaginal specimens and those without (Figure 2).

## Discussion

Studies have demonstrated that semen contains several bioactive molecules with the ability to alter vaginal flora, induce cytokine production, and immune cell recruitment to the FGT after condomless sex (10–13, 17, 18, 25, 48–51). However, few studies investigated the impact of semen exposure on biomarkers of female genital inflammation in relation to HIV acquisition risk. Genital inflammation in women has been linked to an increased susceptibility to HIV infection (2), if semen exposure alters biomarkers of inflammation, then women may be at greater risk of acquiring the virus. Here we demonstrate that semen exposure as measured by YcDNA detection, but not self-report of condom use, had a greater association with biomarkers of epithelial barrier integrity and modulation of BV-associated bacteria than with the cytokine and immune cell responses related to female genital inflammation and HIV risk.

Traditionally, HIV prevention trials and reproductive health studies rely greatly on self-reported data despite acknowledgment of over-reporting (28–30, 52, 53). This study demonstrated a high level of discordance between self-reported condom use and the detection of semen biomarkers in vaginal specimens. In this study, almost a third of the women reporting consistent condom use with their partner had detectable YcDNA in their genital specimens. The challenges associated with inaccurate reporting of condom use among women are established and include: consistency of condom use, incorrect condom use, condom failure, social desirability bias, and recall bias, to name a few (54–60). However, women without detectable YcDNA may either represent those who did use condoms, those who abstained from sex within 15 days, or those who had condomless sex later than 15 days prior to genital sampling. Condom use was over-reported in this study, highlighting the need for routine objective screening for the presence of semen as a biomarker of condomless sex in future HIV prevention studies.

YcDNA detection was associated with marital status, a higher median number of reported vaginal sex acts in the past 30 days, living with or often seeing a partner, and a higher number of lifetime pregnancies compared to YcDNA negative women. The increased presence of semen markers in CVLs from women in stable relationships may be due to several factors, including reduced HIV/STI risk perception and/or an inability to negotiate condom use (61), and late use or early removal of condoms. Additionally, a greater frequency of coital episodes has been associated with increased odds of condomless sex in women (62). A greater number of coital acts with an infected partner may also increase the potential for exposure to sexually transmitted pathogens. Here, gonorrhoeae was associated with YcDNA detection in women. Gonorrhoeae is sexually-transmitted and condomless sexual intercourse with an infected partner is a major risk factor for acquiring the infection (63). However, YcDNA detection was not associated with the other STIs measured, which may be due to a relatively low prevalence of each STI (NG, CT, TV, and MG) in this study. Women with detectable YcDNA in their genital specimens also had a significantly higher median Nugent Score, suggesting that condomless sex is associated with alterations in the vaginal microbiome. These findings are highly consistent with another study reporting that Nugent Scores were significantly associated with the presence of semen in vaginal specimens (64).

Here we investigated the impact of semen exposure on biomarkers of inflammation associated with HIV acquisition in women. YcDNA detection in female genital specimens was used as a biomarker of semen exposure within 15 days of genital sampling (31–33). YcDNA detection at the FGT predicted significantly higher levels of 11/48 cytokines, and with reduced concentrations of two, IL-18, and MIF. Increased concentrations of IL-18 and MIF have previously been implicated in male infertility and reduced sperm motility (65, 66). During reproduction, altered immune responses at the FGT may promote reduced concentrations of these cytokines to facilitate conception. The increase in concentrations of several cytokines is consistent with other studies reporting that semen exposure is associated with cytokine upregulation at the FGT (9, 10, 12, 13, 67). Here, semen exposure was associated with both a pro-inflammatory (IFN-γ, IL-12p70, and IP-10) and anti-inflammatory (IL-10) immune response at the FGT (2, 68, 69). These data support the potential for an initial inflammatory response at the FGT required for embryo implantation and removal of defective sperm, followed by a quick shift to an anti-inflammatory immune response defined by the secretion of IL-10, which may function to promote tolerance to the paternal antigens (14, 15, 25, 70–73). Further, increased concentrations of MIP-1α, MIP-1β, IP-10, and IL-8 have previously been associated with HIV risk in the CAPRISA 004 trial (2). Of these, YcDNA detection was associated only with significant increases in IP-10 in this study, suggesting a limited relationship between semen exposure and those cytokines commonly known to increase the risk of HIV acquisition in women. However, considering that YcDNA is detectable up to 15 days after semen exposure, a biomarker of more recent semen exposure may better characterize the initial pro-inflammatory cytokine response at the FGT which may have implications for HIV risk.

An intact epithelial barrier is a primary host defense against HIV entry and infection. MMPs are proteolytic zinc-dependent enzymes responsible for the degradation and remodeling of the epithelial barrier and have been associated with elevated genital cytokine concentrations (4, 74). YcDNA detection was associated with significant increases in MMP-2 and its regulator TIMP-4. TIMP-4 was likely upregulated at the FGT in response to the high concentrations of MMP-2, since it prevents the activity of MMP-1, MMP-2, MMP-3, MMP-7, and MMP-9 (75, 76). Friction during sexual intercourse has also been associated with microabrasions at the FGT (22, 23). Increased production of MMPs and TIMPs in response to semen exposure and/or friction during condomless sex may compromise the integrity of the female genital epithelial barrier, thereby facilitating HIV entry and access to local target cells. In support of this hypothesis, several studies have demonstrated increased HIV incidence among women with reduced epithelial barrier integrity (77–80). Given that MMPs/TIMPs are only a small subset of proteins that function in maintaining epithelial barrier integrity, further studies are needed using an expanded panel of barrier proteins to reliably assess the impact of condomless sex on the vaginal epithelium.

Recent studies have suggested that vaginal bacteria can also contribute to genital inflammation known to increase HIV risk in women (5, 6). Here, semen exposure was associated with a significantly increased presence of *P. bivia* at the FGT. Semen has an alkaline pH and raises the acidic pH of the vagina to 7.0 or higher after sexual intercourse without a condom, this may favor the growth of BV-associated microbes (20, 81). Additionally, semen also contains a diverse array of microbial communities that have the potential to alter the vaginal microbial composition (17–19). A study conducted in young South African women demonstrated that a diverse vaginal microbiome dominated by anaerobic bacteria was associated with a 4-fold greater risk of acquiring HIV (6). Given that YcDNA detection was associated with an increased presence of *Prevotella*, which has previously been related to HIV risk (6), semen-induced alterations in the vaginal microbiome may have implications for HIV susceptibility in women.

Since HIV requires access to local target cells to establish productive infection, we assessed the impact of YcDNA detection on endocervical T cell frequencies. Here, YcDNA detection was not associated with significant alterations in HIV target cell frequencies at the FGT. This lack of an association between YcDNA detection and endocervical T cell alterations may be due to the longer range of semen detection. Additionally, Th17 cells that are preferential targets for HIV infection (82) and Treg cell populations which may be induced by semen for tolerance to the paternal antigens (16, 83, 84), were not assessed in this study.

The strength of this study lies in the abundance of immunological and microbial data to assess the impact of semen exposure on the FGT in longitudinal analyses. Few studies have investigated the impact of semen exposure at the FGT in the context of HIV. Here, we used a biomarker of semen exposure to reliably assess the impact of condomless sex on multiple biomarkers of inflammation, including those previously associated with HIV risk in women. However, considering potential variations in immune alterations during a period of up to 15 days after semen exposure, comparisons with a biomarker of more recent semen exposure may be required to better assess semen-induced alterations at the FGT. This study was limited by the yield of cervix-derived T cells required to assess both immune activation and regulation, and further investigation is necessary to determine whether YcDNA detection is associated with alterations in endocervical Treg and Th17 cell populations. Here, common BV-associated microbes were assessed using PCR which limits the detection of semen-associated alterations to those specific microbes. The use of 16S rRNA gene sequencing may provide a more comprehensive picture of the impact of semen exposure on the vaginal microbiome. The study was limited in the ability to control for other factors associated with alterations in the immune and microbial environments of the FGT, including the use of vaginal insertive products, menstruation, contraceptive use, etc. Nonetheless, this study demonstrates that semen exposure is associated with immune and microbial changes at the FGT that may have implications for HIV susceptibility in women, and additional studies are required to further characterize these alterations, assess their robustness, and confirm the relative impact on HIV risk.

Here, YcDNA detection, but not self-report of condom use, was associated with shifts in the immune and microbial profiles of the FGT. Although this biomarker of condomless sex <15 days of sampling was not generally associated with the cytokines and immune cells commonly implicated in raised HIV risk, it was, however, associated with biomarkers of epithelial barrier integrity and an increased presence of *P.bivia* which may still have implications for HIV susceptibility in women. This study provides insight into the impact of semen exposure on the FGT and underscores the importance of further studies to better understand the kinetics of these alterations following semen exposure. Taken together, this study emphasizes the reliability of biomarkers of semen exposure over self-report in analyses of female genital immunity and highlights the importance of incorporating biomarkers of semen exposure and controlling for such evidence of condomless sex in future STI/HIV prevention studies. Understanding the specific contribution of semen to a vaginal immune environment conducive to HIV infection may advise the design of targeted biomedical approaches to prevent HIV infection in women.

## Data Availability Statement

The raw data supporting the conclusions of this article will be made available by the authors, without undue reservation.

## Ethics Statement

The studies involving human participants were reviewed and approved by The Biomedical Research Ethics Committee at the University of KwaZulu-Natal. The patients/participants provided their written informed consent to participate in this study.

## Author Contributions

JJ, SN, and LL contributed to the conception and design of the study. JJ, LL, AM, and RS performed the experiments. JJ, LL, and FO analyzed and interpreted the data. JJ, SN, LL, J-AP, QA, LM, and SA wrote the manuscript. All authors contributed to the article and approved the submitted version.

## Conflict of Interest

The authors declare that the research was conducted in the absence of any commercial or financial relationships that could be construed as a potential conflict of interest.
