# Supplementary material for: The Impact of Semen Exposure on the Immune and Microbial Environments of the Female Genital Tract
Source: Front Reprod Health. 2020 Nov 9;2:566559. doi: 10.3389/frph.2020.566559 (PMC9580648; doi:10.3389/frph.2020.566559)
Supplement: Supplementary file 1 [file Data_Sheet_1.docx]

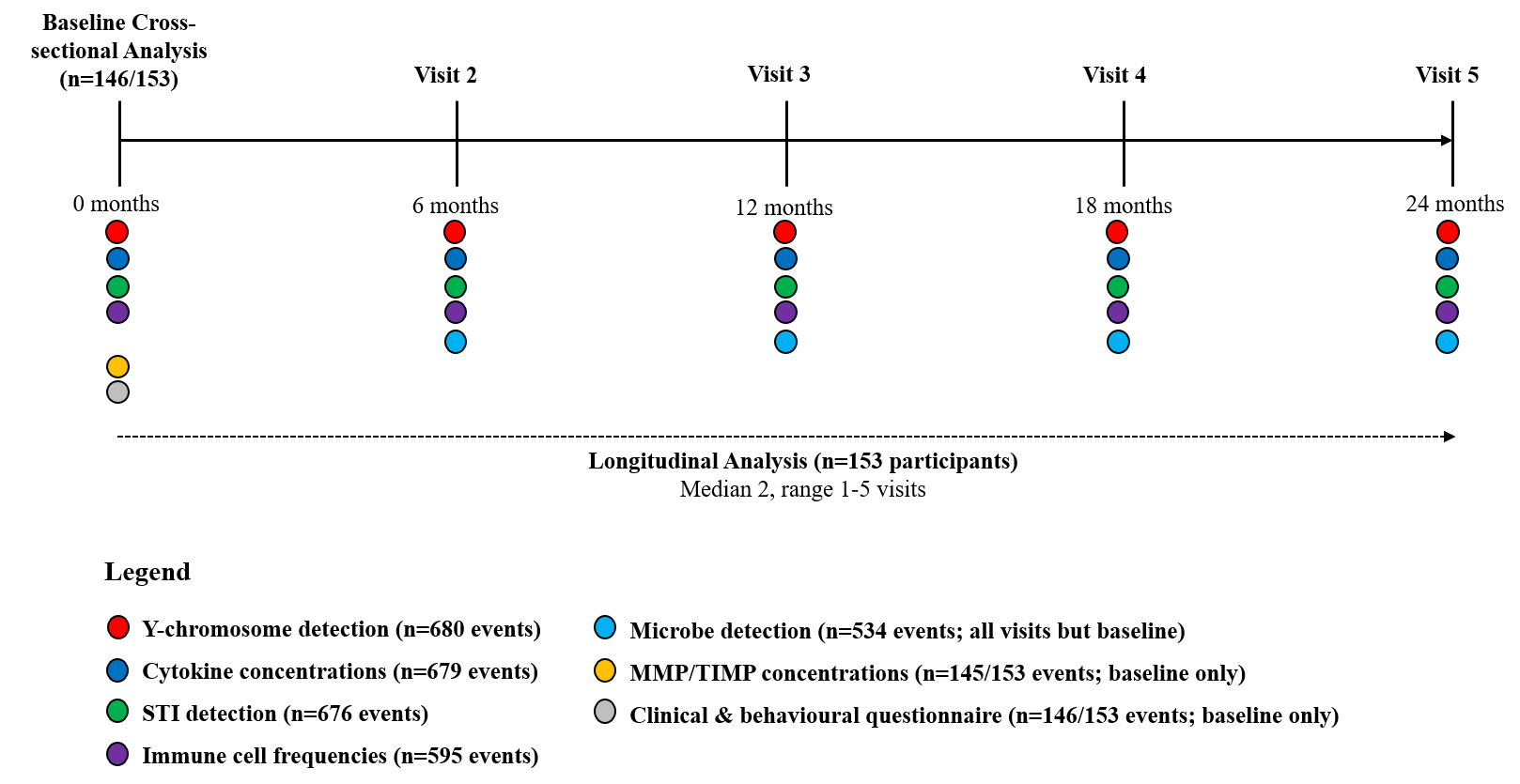


**Supplementary Figure 1.** Graphical representation of the data available at baseline and longitudinally for CAPRISA 008 trial participants. This study included 153 participants from the CAPRISA 008 trial. Genital specimens were collected from participants biannually during the 2-year trial (median 2, range 1-5 visits). The dataset includes all participants with available YcDNA data. Seven of the 153 participants had YcDNA data for all visits but baseline and were therefore excluded from cross-sectional baseline analysis but were included in the longitudinal analyses. MMP/TIMP and questionnaire data were only available at baseline, and microbe PCR data were available at all visits but baseline.


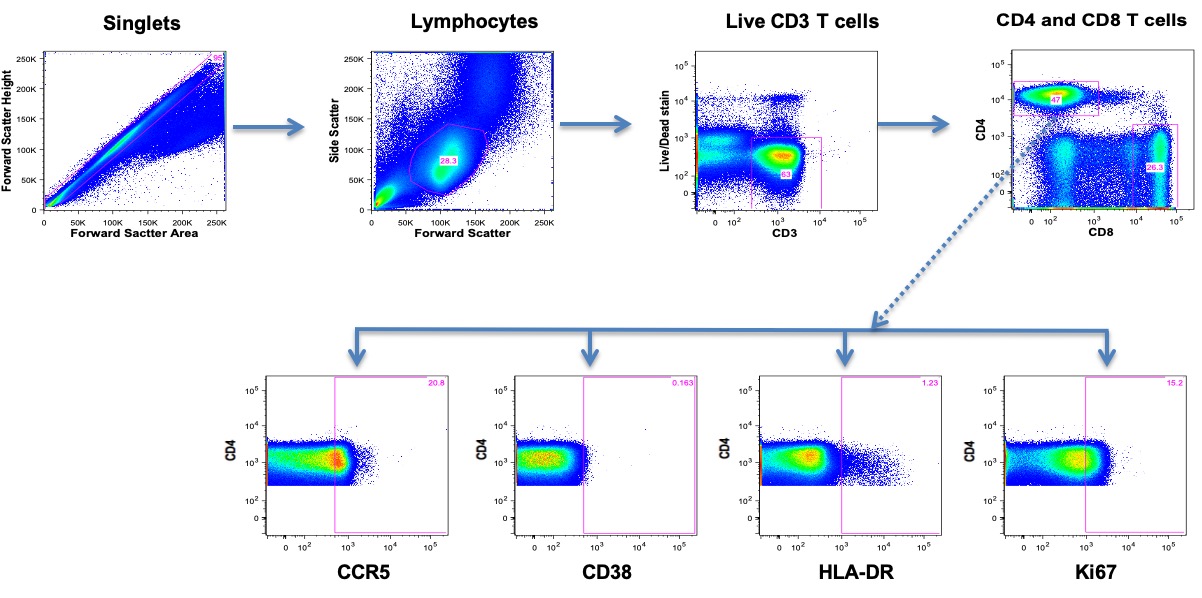


Supplementary Figure 2. Representative flow cytometry gating plot for the assessment of T cell activation. A singlet gate was used to exclude cell doublets. Live cells were identified, followed by lymphocytes, and the CD3+ T cell population. The CD3+ T cell population was divided into CD4+ and CD8+ T cell subsets. The expression of activation markers (CD38+, HLA-DR+), the marker of proliferation (Ki67+), and the HIV co-receptor for entry (CCR5+) was assessed on CD4+ and CD8+ T cells.

**Supplementary Table 1.** Baseline associations between cytokine concentrations and women reporting never using a condom during intercourse with their partner.

|  | **BIVARIABLE** | | | | | **MULTIVARIABLE** | | | |  |
| --- | --- | --- | --- | --- | --- | --- | --- | --- | --- | --- |
|  |  | **95% CI** | |  |  |  | **95% CI** | |  |  |
| **Cytokines** | **β-coefficient** | **Lower** | **Upper** | **P Value** | **FDR** | **β-coefficient** | **Lower** | **Upper** | **P Value** | **FDR** |
| IL-1α | 0.099 | -0.209 | 0.408 | 0.524 | 0.954 | 0.018 | -0.300 | 0.336 | 0.912 | 0.975 |
| IL-1β | 0.061 | -0.393 | 0.516 | 0.788 | 0.954 | -0.060 | -0.501 | 0.380 | 0.785 | 0.975 |
| IL-6 | 0.037 | -0.229 | 0.304 | 0.781 | 0.954 | -0.031 | -0.322 | 0.260 | 0.832 | 0.975 |
| IL-12p40 | 0.011 | -0.348 | 0.371 | 0.950 | 0.954 | -0.006 | -0.417 | 0.404 | 0.975 | 0.975 |
| IL-12p70 | 0.054 | -0.159 | 0.267 | 0.617 | 0.954 | 0.027 | -0.202 | 0.256 | 0.815 | 0.975 |
| IL-18 | 0.194 | -0.162 | 0.551 | 0.280 | 0.954 | 0.147 | -0.227 | 0.521 | 0.436 | 0.975 |
| MIF | 0.241 | -0.154 | 0.635 | 0.228 | 0.954 | 0.155 | -0.251 | 0.561 | 0.448 | 0.975 |
| TNF-α | 0.028 | -0.202 | 0.258 | 0.808 | 0.954 | -0.017 | -0.258 | 0.224 | 0.889 | 0.975 |
| TNF-β | 0.066 | -0.119 | 0.252 | 0.478 | 0.954 | 0.052 | -0.158 | 0.262 | 0.622 | 0.975 |
| TRAIL | 0.110 | -0.216 | 0.435 | 0.504 | 0.954 | 0.049 | -0.282 | 0.379 | 0.769 | 0.975 |
| CTACK | 0.080 | -0.177 | 0.337 | 0.537 | 0.954 | 0.022 | -0.250 | 0.295 | 0.871 | 0.975 |
| EOTAXIN | 0.182 | -0.257 | 0.621 | 0.411 | 0.954 | 0.109 | -0.375 | 0.593 | 0.654 | 0.975 |
| GRO-α | 0.075 | -0.462 | 0.613 | 0.781 | 0.954 | 0.126 | -0.445 | 0.697 | 0.661 | 0.975 |
| IL-8 | -0.047 | -0.401 | 0.308 | 0.793 | 0.954 | -0.070 | -0.450 | 0.310 | 0.714 | 0.975 |
| IL-16 | 0.080 | -0.233 | 0.394 | 0.612 | 0.954 | 0.011 | -0.335 | 0.357 | 0.950 | 0.975 |
| IP-10 | -0.173 | -0.681 | 0.336 | 0.500 | 0.954 | -0.119 | -0.618 | 0.379 | 0.634 | 0.975 |
| MCP-1 | 0.046 | -0.109 | 0.202 | 0.554 | 0.954 | 0.073 | -0.103 | 0.248 | 0.411 | 0.975 |
| MCP-3 | 0.112 | -0.224 | 0.447 | 0.510 | 0.954 | 0.108 | -0.272 | 0.489 | 0.571 | 0.975 |
| MIG | 0.045 | -0.430 | 0.519 | 0.851 | 0.954 | 0.084 | -0.410 | 0.578 | 0.736 | 0.975 |
| MIP-1α | 0.143 | -0.188 | 0.475 | 0.391 | 0.954 | 0.031 | -0.283 | 0.345 | 0.844 | 0.975 |
| MIP-1β | -0.042 | -0.360 | 0.276 | 0.793 | 0.954 | -0.038 | -0.383 | 0.306 | 0.825 | 0.975 |
| RANTES | 0.183 | -0.186 | 0.552 | 0.326 | 0.954 | 0.236 | -0.157 | 0.630 | 0.234 | 0.975 |
| IFN-α2 | 0.067 | -0.102 | 0.236 | 0.429 | 0.954 | 0.049 | -0.138 | 0.237 | 0.600 | 0.975 |
| SDF-1α | 0.145 | -0.139 | 0.428 | 0.312 | 0.954 | 0.108 | -0.201 | 0.418 | 0.488 | 0.975 |
| β-NGF | 0.057 | -0.447 | 0.561 | 0.821 | 0.954 | 0.080 | -0.458 | 0.618 | 0.768 | 0.975 |
| FGF BASIC | -0.003 | -0.059 | 0.054 | 0.928 | 0.954 | -0.022 | -0.078 | 0.034 | 0.433 | 0.975 |
| G-CSF | 0.072 | -0.326 | 0.469 | 0.721 | 0.954 | -0.011 | -0.448 | 0.425 | 0.960 | 0.975 |
| GM-CSF | -0.017 | -0.059 | 0.025 | 0.421 | 0.954 | -0.025 | -0.072 | 0.022 | 0.298 | 0.975 |
| HGF | 0.027 | -0.314 | 0.367 | 0.877 | 0.954 | -0.094 | -0.439 | 0.250 | 0.587 | 0.975 |
| IL-3 | 0.063 | -0.177 | 0.303 | 0.603 | 0.954 | 0.068 | -0.207 | 0.343 | 0.622 | 0.975 |
| IL-7 | 0.102 | -0.154 | 0.358 | 0.431 | 0.954 | -0.008 | -0.224 | 0.208 | 0.942 | 0.975 |
| IL-9 | 0.005 | -0.156 | 0.166 | 0.954 | 0.954 | -0.038 | -0.199 | 0.123 | 0.639 | 0.975 |
| LIF | 0.083 | -0.112 | 0.278 | 0.400 | 0.954 | 0.030 | -0.166 | 0.226 | 0.762 | 0.975 |
| M-CSF | 0.075 | -0.138 | 0.287 | 0.485 | 0.954 | 0.057 | -0.171 | 0.286 | 0.618 | 0.975 |
| PDGF-BB | 0.112 | -0.078 | 0.302 | 0.245 | 0.954 | 0.072 | -0.115 | 0.258 | 0.445 | 0.975 |
| SCF | 0.026 | -0.344 | 0.395 | 0.891 | 0.954 | 0.036 | -0.350 | 0.422 | 0.853 | 0.975 |
| SCGF-β | 0.044 | -0.343 | 0.431 | 0.822 | 0.954 | -0.049 | -0.463 | 0.365 | 0.815 | 0.975 |
| VEGF | 0.048 | -0.230 | 0.326 | 0.730 | 0.954 | 0.012 | -0.288 | 0.312 | 0.936 | 0.975 |
| IFN-γ | 0.036 | -0.149 | 0.221 | 0.701 | 0.954 | -0.023 | -0.210 | 0.165 | 0.809 | 0.975 |
| IL-2 | 0.128 | -0.328 | 0.585 | 0.577 | 0.954 | 0.095 | -0.422 | 0.611 | 0.716 | 0.975 |
| IL-4 | 0.024 | -0.136 | 0.184 | 0.763 | 0.954 | -0.040 | -0.173 | 0.093 | 0.554 | 0.975 |
| IL-5 | 0.336 | -0.042 | 0.715 | 0.081 | 0.954 | 0.321 | -0.075 | 0.717 | 0.110 | 0.975 |
| IL-13 | 0.083 | -0.088 | 0.255 | 0.336 | 0.954 | 0.033 | -0.137 | 0.203 | 0.698 | 0.975 |
| IL-15 | 0.219 | -0.281 | 0.720 | 0.385 | 0.954 | 0.323 | -0.232 | 0.877 | 0.249 | 0.975 |
| IL-17A | -0.013 | -0.164 | 0.138 | 0.863 | 0.954 | -0.036 | -0.196 | 0.125 | 0.657 | 0.975 |
| IL-2rα | 0.005 | -0.176 | 0.186 | 0.954 | 0.954 | -0.017 | -0.210 | 0.177 | 0.862 | 0.975 |
| IL-10 | 0.024 | -0.094 | 0.143 | 0.683 | 0.954 | -0.002 | -0.123 | 0.118 | 0.972 | 0.975 |
| IL-1RA | 0.034 | -0.123 | 0.190 | 0.668 | 0.954 | 0.029 | -0.130 | 0.188 | 0.715 | 0.975 |
| β-coefficients and corresponding P-values were determined using linear regression models. Bivariable regression models were adjusted for randomization arm. Multivariable regression models were adjusted for age, any STI (*C. trachomatis. N. gonorrhoea. T. vaginalis. M. genitalium*), Nugent Score, number of vaginal sex acts in the past 30 days, and randomization arm. | | | | | | | | | | |

**Supplementary Table 2.** Baseline associations between MMP/TIMP concentrations and women reporting never using a condom during intercourse with their partner.

|  | **BIVARIABLE** | | | | | **MULTIVARIABLE** | | | |  |
| --- | --- | --- | --- | --- | --- | --- | --- | --- | --- | --- |
|  |  | **95% CI** | |  |  |  | **95% CI** | |  |  |
| **MMP/ TIMP** | **β-coefficient** | **Lower** | **Upper** | **P Value** | **FDR** | **β-coefficient** | **Lower** | **Upper** | **P Value** | **FDR** |
| MMP-1 | 0.148 | -0.338 | 0.634 | 0.545 | 0.796 | 0.067 | -0.444 | 0.578 | 0.793 | 0.836 |
| MMP-2 | 0.275 | -0.294 | 0.844 | 0.338 | 0.796 | 0.239 | -0.270 | 0.749 | 0.352 | 0.836 |
| MMP-3 | 0.103 | -0.241 | 0.447 | 0.551 | 0.796 | 0.129 | -0.217 | 0.475 | 0.458 | 0.836 |
| MMP-7 | 0.040 | -0.488 | 0.568 | 0.882 | 0.891 | -0.119 | -0.699 | 0.462 | 0.684 | 0.836 |
| MMP-8 | -0.584 | -1.897 | 0.729 | 0.378 | 0.796 | -0.791 | -2.013 | 0.432 | 0.201 | 0.836 |
| MMP-9 | -0.172 | -0.859 | 0.515 | 0.619 | 0.796 | -0.332 | -0.889 | 0.225 | 0.238 | 0.836 |
| MMP-10 | 0.202 | -0.291 | 0.695 | 0.416 | 0.796 | 0.103 | -0.406 | 0.612 | 0.687 | 0.836 |
| MMP-12 | -0.175 | -0.720 | 0.371 | 0.525 | 0.796 | -0.051 | -0.536 | 0.435 | 0.836 | 0.836 |
| MMP-13 | -0.033 | -0.513 | 0.447 | 0.891 | 0.891 | -0.121 | -0.600 | 0.358 | 0.615 | 0.836 |
| TIMP-1 | 0.182 | -0.086 | 0.451 | 0.180 | 0.360 | 0.216 | -0.058 | 0.491 | 0.120 | 0.240 |
| TIMP-2 | -0.058 | -0.305 | 0.190 | 0.643 | 0.643 | -0.025 | -0.293 | 0.242 | 0.850 | 0.850 |
| TIMP-3 | 0.212 | -0.090 | 0.515 | 0.166 | 0.360 | 0.263 | -0.069 | 0.596 | 0.118 | 0.240 |
| TIMP-4 | 0.189 | -0.240 | 0.618 | 0.383 | 0.511 | 0.206 | -0.257 | 0.668 | 0.378 | 0.504 |
| β-coefficients and corresponding P-values were determined using linear regression models. Bivariable regression models were adjusted for randomization arm. Multivariable regression models were adjusted for age, any STI (*C. trachomatis, N. gonorrhoea, T. vaginalis, M. genitalium*), Nugent Score, number of vaginal sex acts in the past 30 days, inflammation status, and randomization arm. | | | | | | | | | | |

**Supplementary Table 3.** Baseline associations between immune cell frequencies and women reporting never using a condom during intercourse with their partner.

|  | **BIVARIABLE** | | | |  | **MULTIVARIABLE** | | | |  |
| --- | --- | --- | --- | --- | --- | --- | --- | --- | --- | --- |
|  |  | **95% CI** | |  |  |  | **95% CI** | |  |  |
| **Immune cell subsets** | **β-coefficient** | **Lower** | **Upper** | **P value** | **FDR** | **β-coefficient** | **Lower** | **Upper** | **P value** | **FDR** |
| CD4+ | -0.051 | -0.132 | 0.029 | 0.209 | 0.502 | -0.042 | -0.136 | 0.051 | 0.370 | 0.757 |
| CD4+CCR5+ | 0.137 | 0.001 | 0.273 | 0.049 | 0.444 | 0.118 | -0.029 | 0.265 | 0.113 | 0.639 |
| CD4+CCR5+CD38+ | 0.070 | -0.027 | 0.168 | 0.152 | 0.489 | 0.072 | -0.034 | 0.179 | 0.180 | 0.639 |
| CD4+CCR5+HLA-DR+ | 0.081 | -0.033 | 0.195 | 0.163 | 0.489 | 0.075 | -0.044 | 0.195 | 0.213 | 0.639 |
| CD4+CCR5+KI67+ | 0.059 | -0.102 | 0.220 | 0.465 | 0.620 | 0.044 | -0.116 | 0.205 | 0.578 | 0.757 |
| CD4+CD38+ | -0.012 | -0.130 | 0.105 | 0.834 | 0.834 | -0.008 | -0.133 | 0.117 | 0.896 | 0.896 |
| CD4+CD38+HLA-DR+ | 0.037 | -0.039 | 0.112 | 0.335 | 0.620 | 0.034 | -0.051 | 0.120 | 0.424 | 0.757 |
| CD4+CD38+HLA-DR+CCR5+ | 0.060 | -0.006 | 0.126 | 0.074 | 0.444 | 0.056 | -0.019 | 0.131 | 0.138 | 0.639 |
| CD4+KI67+ | 0.052 | -0.120 | 0.224 | 0.545 | 0.654 | 0.036 | -0.149 | 0.222 | 0.694 | 0.757 |
| CD4+HLA-DR+ | 0.031 | -0.088 | 0.150 | 0.605 | 0.660 | 0.026 | -0.100 | 0.151 | 0.683 | 0.757 |
| CD4 Total Activation**^1^** | 0.037 | -0.055 | 0.128 | 0.424 | 0.620 | 0.020 | -0.081 | 0.121 | 0.694 | 0.757 |
| CD8+ | 0.021 | -0.059 | 0.101 | 0.602 | 0.923 | 0.010 | -0.081 | 0.101 | 0.828 | 0.963 |
| CD8+CCR5+ | 0.055 | -0.079 | 0.189 | 0.415 | 0.923 | 0.068 | -0.079 | 0.216 | 0.359 | 0.963 |
| CD8+CD38+ | 0.013 | -0.117 | 0.142 | 0.846 | 0.923 | 0.010 | -0.136 | 0.156 | 0.891 | 0.963 |
| CD8+HLA-DR+ | -0.001 | -0.148 | 0.145 | 0.988 | 0.988 | -0.008 | -0.170 | 0.153 | 0.917 | 0.963 |
| CD8+CD38+HLA-DR+ | 0.010 | -0.092 | 0.113 | 0.840 | 0.923 | 0.004 | -0.113 | 0.120 | 0.948 | 0.963 |
| CD8+KI67+ | 0.127 | -0.070 | 0.324 | 0.200 | 0.923 | 0.070 | -0.145 | 0.284 | 0.515 | 0.963 |
| CD8 Total Activation**^1^** | 0.011 | -0.088 | 0.110 | 0.830 | 0.923 | -0.003 | -0.114 | 0.108 | 0.963 | 0.963 |
| β-coefficients and corresponding P-values were determined using linear regression models. Bivariable regression models were adjusted for randomization arm. Multivariable regression models were adjusted for age, any STI (*C. trachomatis, N. gonorrhoea, T. vaginalis, M. genitalium*), Nugent Score, number of vaginal sex acts in the past 30 days, inflammation status, and randomization arm. **^1^**Total activation refers to cells expressing CCR5, HLA-DR, KI67 and/or CD38. | | | | | | | | | | |
